# Expression of cytokeratin 20 in thyroid carcinomas and peripheral blood detected by reverse transcription polymerase chain reaction

**DOI:** 10.1054/bjoc.1999.0893

**Published:** 1999-12-08

**Authors:** T Weber, J Lacroix, J Weitz, K Amnan, A Magener, T Hölting, E Klar, C Herfarth, M von Knebel Doeberitz

**Affiliations:** Division for Molecular Diagnostics and Therapy Department of Pathology Department of Surgery, University of Heidelberg, Im Neuenheimer Feld 110, Heidelberg, 69120, Germany

**Keywords:** thyroid carcinoma, cytokeratin 20, circulating tumour cells, polymerase chain reaction

## Abstract

We investigated a nested reverse transcriptase polymerase chain reaction (RT-PCR) system to detect CK20 mRNA in thyroid carcinomas, benign thyroid diseases and peripheral blood to improve diagnosis of thyroid carcinoma and to detect disseminated tumour cells. Frozen tissue samples of 46 thyroid carcinomas and 30 benign thyroid diseases (14 multinodular goiters, 14 follicular adenomas, two Hashimoto's hyroiditis) were obtained intraoperatively. Preoperative blood samples were drawn from 31 patients with thyroid cancer, nine patients with benign thyroid disorders and 20 healthy volunteers. Nine out of nine medullary, 9/12 follicular, 7/19 papillary and 2/6 anaplastic carcinomas expressed CK20 transcripts. CK20 mRNA was undetectable in 30 tissue sections of benign thyroid diseases. Circulating tumour cells were found in the blood of 3/8 patients with medullary carcinoma, 2/8 patients with follicular carcinoma, 2/11 patients with papillary carcinoma and 1/4 patients with an anaplastic carcinoma. Nine blood samples of patients with benign thyroid diseases and 20 healthy volunteers tested negative. For the first time CK20 mRNA could be detected in tissue sections of thyroid carcinomas and peripheral blood samples of patients with thyroid cancer. It was not detectable in benign thyroid diseases. Our results therefore strongly suggest that CK20 RT-PCR assays may improve the diagnosis of thyroid carcinoma and is able to detect circulating tumour cells in peripheral blood of thyroid carcinoma patients. © 2000 Cancer Research Campaign


*British Journal of Cancer* (2000) **82**(1), 157–160
© 2000 Cancer Research Campaign
Article no. bjoc.1999.0893

### Expression of cytokeratin 20 in thyroid carcinomas and peripheral blood detected by reverse transcription polymerase chain reaction

**T Weber[1], J Lacroix[1], J Weitz[1], K Amnan[2], A Magener[2], T Hölting[3], E Klar[3], C Herfarth[3] and M von Knebel Doeberitz[1]**

[1]Division for Molecular Diagnostics and Therapy, [2]Department of Pathology and [3]Department of Surgery, University of Heidelberg, Im Neuenheimer Feld 110, 69120 Heidelberg, Germany

**Summary** We investigated a nested reverse transcriptase polymerase chain reaction (RT-PCR) system to detect CK20 mRNA in thyroid carcinomas, benign thyroid diseases and peripheral blood to improve diagnosis of thyroid carcinoma and to detect disseminated tumour cells. Frozen tissue samples of 46 thyroid carcinomas and 30 benign thyroid diseases (14 multinodular goiters, 14 follicular adenomas, two Hashimoto's thyroiditis) were obtained intraoperatively. Preoperative blood samples were drawn from 31 patients with thyroid cancer, nine patients with benign thyroid disorders and 20 healthy volunteers. Nine out of nine medullary, 9/12 follicular, 7/19 papillary and 2/6 anaplastic carcinomas expressed CK20 transcripts. CK20 mRNA was undetectable in 30 tissue sections of benign thyroid diseases. Circulating tumour cells were found in the blood of 3/8 patients with medullary carcinoma, 2/8 patients with follicular carcinoma, 2/11 patients with papillary carcinoma and 1/4 patients with an anaplastic carcinoma. Nine blood samples of patients with benign thyroid diseases and 20 healthy volunteers tested negative. For the first time CK20 mRNA could be detected in tissue sections of thyroid carcinomas and peripheral blood samples of patients with thyroid cancer. It was not detectable in benign thyroid diseases. Our results therefore strongly suggest that CK20 RT-PCR assays may improve the diagnosis of thyroid carcinoma and is able to detect circulating tumour cells in peripheral blood of thyroid carcinoma patients. © 2000 Cancer Research Campaign

**Keywords**: thyroid carcinoma; cytokeratin 20; circulating tumour cells; polymerase chain reaction

Clinically palpable thyroid nodules occur in 4–7% (Gharib, 1994; Tezelman and Clark, 1995) of the population in the USA. A German study showed that, in areas of iodine deficiency, thyroid nodules are detected in up to 20% (Raue, 1995). In another study, the incidence of thyroid carcinoma ranged from 0.5 to 10 cases per 100 000 population (Schlumberger, 1998).

Thyroid cancers arise from follicular cells (papillary, follicular and anaplastic carcinoma) and parafollicular epithelium (medullary carcinoma). Diagnosis of thyroid carcinoma remains difficult. The best method of distinguishing between benign and malignant thyroid nodules, essential for operative decision-making, is fine-needle aspiration cytology (Gharib, 1994; Tezelman and Clark, 1995; Schlumberger, 1998). The major problem of this procedure is the impossibility of differentiation between follicular adenoma and follicular carcinoma because capsular or venous tumour invasion cannot be detected.

At our department, 90 patients with papillary, 73 with follicular, 20 with anaplastic and 108 with medullary carcinoma of the thyroid were treated surgically from 1987 until July 1998. In a retrospective study (Weber et al, 1997) we demonstrated that a correct preoperative diagnosis of thyroid cancer could only be reached in 40% of these cases.

Moll et al (1990) described an intermediate filament group protein cytokeratin 20 (CK20), contributing to differentiation and function of the cytoskeleton of mucosal epithelia of the mammalian gastrointestinal tract (Moll et al, 1992, 1993). In an immunhistochemical analysis of 711 cases of primary and metastatic cancer, CK20 was observed in adenocarcinomas of colon and rectum, stomach, bile system and pancreas, ovarian tumours, transitional-cell and Merkel-cell carcinomas (Moll et al, 1992). Papillary ($n$ = 2) and follicular ($n$ = 4) thyroid carcinomas showed no expression of CK20. Medullary carcinoma stained positive with CK20 antibodies (Moll et al, 1992).

In 1996, Schröder et al published an immunhistochemical study in which he found an expression of CK20 in two of 27 medullary carcinomas but no reactivity in papillary, follicular and anaplastic thyroid carcinomas.

Polymerase chain reaction (PCR) increased the sensitivity of the detection of circulating tumour cells and micrometastases in peripheral blood up to 1 tumour cell in $10^6$ normal cells (Ghossein and Rosai, 1996; Gunn et al, 1996). Burchill et al 1995 was the first to use CK20 reverse transcription PCR (RT-PCR) assays to amplify CK20 transcripts as evidence for circulating tumour cells in peripheral blood and bone marrow of patients with colorectal cancer.

In this study we used a recently described CK20 RT-PCR protocol (Weitz et al, 1998) to amplify CK20 transcripts from thyroid carcinoma cell lines, frozen tissue sections of benign and malignant thyroid diseases and peripheral blood of patients with thyroid cancer in order to improve diagnosis of thyroid carcinoma and to detect circulating tumour cells.

*Received 4 May 1999*
*Revised 14 July 1999*
*Accepted 15 July 1999*

*Correspondence to:* T Weber

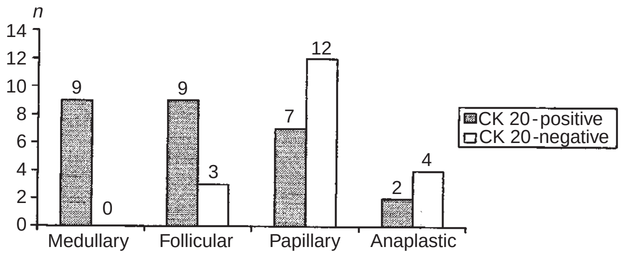

**Figure 1** Detection of CK20 mRNA in thyroid carcinoma tissue (*n* = 46)

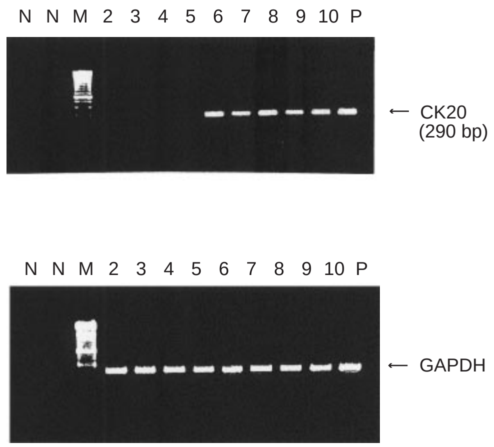

**Figure 2** CK20 RT-PCR amplification products in tissue sections of papillary, follicular, medullary and anaplastic thyroid carcinoma. N, negative controls; M, molecular weight marker (100 bp); P, positive control (TT cell line). Lanes 2–4, no detection of CK20 mRNA in 3 papillary carcinomas; lanes 5–7, CK20 amplification in two of three follicular carcinomas; lanes 8–10, positive results for three of three medullary carcinomas

### PATIENTS AND METHODS

### Cell lines

Four well characterized thyroid carcinoma cell lines were used in the study. They included the follicular carcinoma cell line FTC 133, the medullary cell line TT and two anaplastic cell lines SW 1736 and C 643.

### Patient samples

Informed consent was obtained from all patients, and the study protocol was approved by the ethics committee of the University of Heidelberg. In case of suspected thyroid cancer, biopsy specimens from consenting patients with benign and malignant thyroid diseases were obtained intraoperatively during subtotal or total thyroidectomy at the Department of Surgery of the University of Heidelberg, immediately snap-frozen in liquid nitrogen and stored at –70°C.

Peripheral blood samples (10 ml, EDTA) were drawn from healthy volunteers and preoperatively from patients undergoing surgery for benign and malignant thyroid diseases. Patients with known secondary malignancies were excluded from the study.

After dilution with 10 ml phosphate-buffered saline (PBS) and density centrifugation through Ficoll-Paque (Pharmacia), mononuclear blood cells were harvested and washed twice in PBS. The cell pellet was snap-frozen in liquid nitrogen and stored at –70°C.

### RNA extraction

Total RNA from cell lines, frozen tissue sections of benign thyroid diseases and thyroid carcinomas and mononuclear blood cells was extracted using a commercially available RNA extraction kit (Trizol Reagent, Life Technologies, Inc., Karlsruhe, Germany).

### RT-PCR

CK20 RT-PCR was essentially performed as described by Weitz et al (1998). Briefly, cDNA was synthesized using a reverse transcription kit (Life Technologies, Inc.) and the primer CK20 558.rev.

PCR was performed in 50-μl samples that contained a final concentration of 25 pmol each primer, 0.2 mM each NTP, 1.5 mM magnesium chloride, 2.5 units *Taq* polymerase and 20 mM PCR buffer (PCR kit: Life Technologies, Inc.). For the first PCR 2.5 μl cDNA was used to amplify CK20 cDNAs with the primers 1.for (ATGGATTTCAGTCGCAGA) and 558.rev (ATGTAGGGTTAGGTCATCAAAG). Thirty-five rounds of amplification were performed at 30-s intervals at temperatures of 93°C, 60°C and 72°C. The nested PCR was performed with 10 μl PCR product of the first PCR with primer 139.for (TCCAACTCCAGACACACGGTGAACTATG) and 429.rev (CAGGACACACCGAGCATTTTGCAG) under the amplification conditions as described for the first PCR with an annealing temperature of 72°C. PCR products were analysed by electrophoresis on 2% agarose gels. In addition, direct thermocycle sequencing and in situ hybridization of the obtained amplimere was performed.

### Sensitivity

The sensitivity of the CK20 RT-PCR assay was determined in dilution experiments using the medullary thyroid carcinoma cell line TT and the colon cancer cell line HT29. Tumour cells (0, 10, $10^2$, $10^3$, $10^4$, $10^5$) were added to 10 ml of peripheral blood samples of healthy donors, mRNA extracted and RT-PCR was performed for CK20.

### Northern blot mRNA was separated on 0.8% MOPS agarose gels and blotted to Hybond N Plus membranes. PolyA Plus mRNA was transferred to Hybond N Plus (Amersham, Little Chalfont, UK) membranes. Nested CK20 PCR products were labelled with (Alpha-$^{32}$P)-dCTP using the random primed cDNA labelling kit from MBI Fermentas (Amherst, NY, USA). These filters were hybridized with labelled CK20 probes according to standard protocols.

### RESULTS

### Sensitivity of CK20 RT-PCR

A 290-bp PCR product was amplified from samples enriched with TT cells up to a dilution of $10^5$ TT cells in 10-ml peripheral blood. As previously described (Weitz et al, 1998) CK20 transcripts from blood samples enriched with HT 29 cells, a colon carcinoma cell line, could be detected up to a dilution of ten HT 29 cells in 10 ml blood. Direct thermocycle sequencing confirmed that the 290-bp amplimere corresponded to amplified CK20 cDNA fragments.

*© 2000 Cancer Research Campaign*

**Table 1** Expression of CK20 mRNA in preoperative blood samples of patients with thyroid carcinoma (*n* = 31)

| Histology | Positive results | |
|---|---|---|
| Medullary carcinoma | 3/8 | (38%) |
| Follicular carcinoma | 2/8 | (25%) |
| Papillary carcinoma | 2/11 | (18%) |
| Anaplastic carcinoma | 1/4 | (25%) |
| Total | 8/31 | (26%) |

**Table 2** Detection of circulating tumour cells in peripheral blood of patients with medullary thyroid carcinomas (*n* = 8)

| Stage (TNM) | CK20-positive |
|---|---|
| T1 | 0/3 |
| T2 | – |
| T3 | 0/1 |
| T4 | 1/2 |
| Local recurrence | 2/2 |

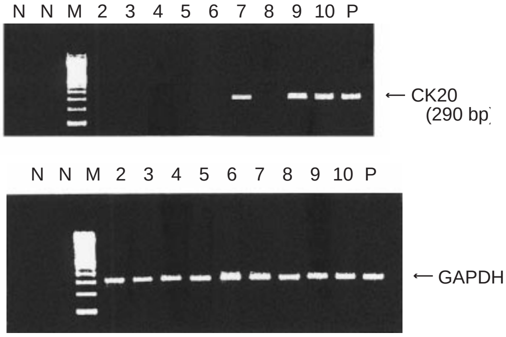

**Figure 3** Detection of CK20 mRNA in preoperative blood samples of patients with thyroid carcinoma. N, negative contrils; M, molecular weight market (100 bp); P, positive control (TT cell line). Lanes 2–4, no detection of CK20 mRNA in three blood samples of patients with papillary carcinoma; Lanes 5–7, detection in CK20 in one of three patients with follicular carcinoma; Lanes 8–10, detection of CK20 in two of three patients with medullary carcinoma

A Northern blot analysis was performed to compare the levels of CK20 mRNA expression in TT and HT 29 cells quantitatively. A significantly stronger CK20 hybridization signal was obtained in the colon carcinoma cell line HT 29 compared to the medullary carcinoma cell line TT could be demonstrated.

### Specifity of CK20 RT-PCR

In line with previous reports CK20 RT-PCR products could not be detected in 20 of 20 control blood samples from healthy volunteers. The integrity of mononuclear blood cell RNA was confirmed by RT-PCR for glyceraldehycle 3-phosphate dehydrogenase (GAPDH).

### Thyroid carcinoma cell lines

Three (TT, SW 1736, C 643) of four human thyroid carcinoma cell lines expressed CK20 mRNA, as demonstrated by amplification of the 290-bp CK20 transcript. The follicular carcinoma cell line FTC 133 tested negative.

### Benign and malignant thyroid tissue

CK20 mRNA was undetectable in 30 benign thyroid tissue sections (14 multinodular goiters, 14 follicular adenomas and two cases of Hashimoto's thyroiditis). CK20 transcripts were amplified in 9/9 (100%) medullary carcinomas, 9/12 (75%) follicular carcinomas, 7/19 (37%) papillary thyroid carcinomas and 2/6 (33%) anaplastic carcinomas (Figures 1 and 2).

### Peripheral blood samples

Nine out of nine blood samples of patients with benign thyroid diseases (five multinodular goiters, four follicular adenomas) tested negative for CK20 RT-PCR.

Peripheral blood samples could be obtained from 31 of 46 patients with thyroid cancer. CK20 mRNA could not be detected in 12 blood samples which were obtained from 19 patients whose tumours did not express CK20 transcripts.

Eighteen blood samples were derived from 27 patients with carcinomas positive in the CK20 RT-PCR assay. Due to the histological classification of the tumours the following results could be demonstrated: CK20 mRNA was detected in peripheral blood samples of 3/8 patients with medullary carcinoma, 2/6 with follicular carcinoma 2/4 with papillary thyroid carcinoma and one patient with an anaplastic carcinoma (Table 1). In the cases of medullary carcinoma the CK20 mRNA-positive samples were obtained from one patient with an advanced medullary carcinoma staged pT4N1bM0 and two patients with local recurrence of medullary thyroid cancer. In the two blood samples of patients with follicular carcinomas and detectable CK20 transcripts the tumour stages were classified histologically as pT2N0M0 and pT4N1bM1 (lung metastases). In case of papillary carcinoma the blood samples tested positive for CK20 mRNA were obtained from a patient with a pT2N0M0 tumour and a patient with local recurrence (Table 2).

### DISCUSSION

According to the literature a number of thyroid-specific tumour markers such as thyroglobulin (Ringel et al, 1998; Tallini et al, 1998; Weber et al, 1998), thyroid peroxidase (Tallini et al, 1998) and the human sodium-iodine symporter (Smanik et al, 1997) were investigated in order to establish a molecular diagnosis of thyroid carcinoma. However, the common problem of all of these PCR systems was a high rate of false-positive results. RNA of thyroglobulin (Ringel et al, 1998; Weber et al, 1998), thyroid peroxidase and the sodium-iodine symporter (unpublished data) was detected in blood samples of healthy volunteers. Recently, Ringel et al (1998) demonstrated that normal thyroid cells can be detected in peripheral blood samples. The concept of our study therefore was not to establish a thyroid-specific marker, but a tumour-specific marker, which is expressed by thyroid carcinoma cells because of a different gene expression in malignant transformed cells.

The detection of circulating tumour cells with CK20 RT-PCR in peripheral blood and bone marrow of patients with primary carcinomas and their metastases such as colorectal cancer (Burchill et al, 1995; Gunn et al, 1996; Soeth et al, 1997; Wertz et al, 1998; Wyld et al, 1998), stomach cancer (Soeth et al, 1997), pancreatic cancer (Soeth et al, 1997) and bladder carcinoma (Klein

*© 2000 Cancer Research Campaign* 

et al, 1998) has created a new way of understanding the mechanisms of recurrent disease. Nevertheless, the correlation between tumour progression or prognosis is still controversial. While Wyld et al (1998) could not find a correlation for patients with colorectal cancer, Soeth et al (1997) described a statistically proven shorter survival for patients with colorectal and stomach cancer who tested positive for CK20 mRNA in blood and bone marrow samples. Investigations of cytokeratin markers in thyroid carcinomas have been published by Moll et al (1992) and Schröder et al (1996). Using an immunhistochemical assay they found an expression of CK20 in 1/1 (Moll et al, 1992) and 2/27 (Schröder et al, 1996) medullary carcinomas respectively.

Since the sensitivity of RT-PCR in detecting CK20 mRNA is much higher, we established this method to investigate thyroid tumours. We demonstrated an expression of CK20 mRNA in a medullary (TT) and two anaplastic (SW 1736, C 643) thyroid carcinoma cell lines. Compared to the colon carcinoma cell line HT29, TT cells, however, showed a weaker expression of CK20 in a Northern blot examination.

CK20 mRNA was detected in primary and recurrent thyroid carcinomas depending on the histological classification of the tumours. All of the nine medullary carcinomas, 9/12 follicular carcinomas, 7/19 papillary carcinomas and 2/6 anaplastic carcinomas showed an amplification of CK20 transcripts. In one case a papillary carcinoma (pT4N0M0) tested negative for the primary tumour and positive for the recurrent disease, which occurred 1 year after total thyroidectomy. An explanation for this obvious difference in expressing CK20 may be a shift of the tumour into a lower grade of differentiation and a greater transcriptional relaxation of the recurrent tumour.

To detect circulating tumour cells, we examined peripheral blood samples obtained from 19 of 27 patients with thyroid carcinomas tested positive for CK20. An expression of CK20 mRNA in peripheral blood could be demonstrated in three of eight cases of medullary carcinoma, two of six patients with follicular carcinomas, two of four patients with papillary carcinomas and one patient with an anaplastic carcinoma. The sensitivity for detecting CK20 mRNA in peripheral blood of patients with CK20-positive carcinomas was 42% (8/19 blood samples tested positive), the sensitivity for all tested blood samples was 26% (8/31). Since the number of examined blood samples divided into four different histological types of thyroid carcinoma is relatively small, a correlation between the detection of circulating tumour cells and the stage of the disease is only shown for medullary carcinomas (Table 2).

CK20 mRNA was undetectable in 30 tissue sections of multinodular goiters, follicular adenomas and Hashimoto's thyroiditis. Twenty blood samples from healthy volunteers, nine blood samples of patients with benign thyroid diseases and 12 blood samples of patients with CK20-negative carcinomas tested consistently negative in CK20 PCR. Since there were no false-positive results, the specificity reached 100%.

We hypothesized that CK20 RT-PCR may contribute in improving preoperative diagnosis of thyroid cancer. Our results showed for the first time that CK20 mRNA can be detected in thyroid carcinoma tissue and preoperative blood samples of cancer patients but not in tissue and blood samples of patients with benign thyroid disorders. These preliminary results and their clinical value will be continuously evaluated on a larger group of patients, observed over a longer period of time.

### ACKNOWLEDGEMENTS

We wish to express our thanks to Prof. Dr Uwe Haberkorn, Department of Nuclear Medicine, University of Heidelberg for providing us with anaplastic thyroid carcinoma cell lines SW 1746 and C 643 and Prof. Dr Peter E Goretzki, Surgical Department, University of Düsseldorf for the follicular cell line FTC 133. In addition, we thank Sigrid Weichert and Andrea Schrödel for their excellent technical assistance.

### REFERENCES

Burchill SA, Bradbury MF, Pittman K, Southgate J, Smith B and Selby P (1995) Detection of epithelial cancer cells in peripheral blood by reverse transcriptase – polymerase chain reaction. *Br J Cancer* **71**: 278–281

Gharib H (1994) Fine-needle aspiration biopsy of thyroid nodules: advantages, limitations and effect. *Mayo Clin Proc* **69**: 44–49

Ghossein RA and Rosai J (1996) Polymerase chain reaction in the detection of micrometastases and circulating tumor cells. *Cancer* **78**: 10–16

Gunn J, McCall JL, Yun K and Wright PA (1996) Detection of micrometastases in colorectal cancer patients by K19 and K20 reverse-transcription polymerase chain reaction. *Lab Invest* **75**: 611–616

Johnson PWM, Burchill SA and Selby PJ (1995) The molecular detection of circulating tumor cells. *Br J Cancer* **72**: 268–276

Klein A, Zemer R, Buchumensky V, Klaper R and Nissenkorn I (1998) Expression of cytokeratin 20 in urinary cytology of patients with bladder carcinoma. *Cancer* **82**: 349–354

Moll R, Schiller DL and Franke WW (1990) Identification of protein IT of the intestinal cytoskeleton as a novel type I cytokeratin with unusual properties and expression patterns. *J Cell Biol* **111**: 567–580

Moll R, Löwe A, Laufer J and Franke WW (1992) Cytokeratin 20 in human carcinomas. *Am J Pathol* **140**: 427–447

Moll R, Zimbelmann R, Goldschmidt MD, Keith M, Laufer J, Kasper M, Koch PJ and Franke WW (1993) The human gene encoding cytokeratin 20 and ist expression during fetal development and in gastrointestinal carcinomas. *Differentiation* **53**: 75–93

Raue F (1995) Struma maligna. *Nuklearmed* **3**: 147–152

Ringel MD, Ladenson PW and Levine MA (1998) Molecular diagnosis of residual and recurrent thyroid cancer by amplification of thyroglobulin messenger ribonucleic acid in peripheral blood. *J Clin Endocrinol Metab* **83**: 4435–4442

Schlumberger MJ (1998) Papillary and follicular thyroid carcinoma. *N Engl J Med* **338**: 297–306

Schröder S, Wodzynski A and Padberg B (1996) Zytokeratinexpression benigner und maligner epithelialer Schilddrüsentumoren. *Pathologe* **17**: 425–432

Smanik PA, Ryu KY, Theil KS, Mazzaferri EL and Jhiang SM (1997) Expression, exonintron organization, and chromosome mapping of the human sodium iodine symporter. *Endocrinology* **138**: 3555–3558

Soeth E, Vogel I, Röder C, Juhl H, Marxsen J, Krüger U, Henne-Bruns D, Kremer B and Kalthoff H (1997) Comparative analysis of bone marrow and venous blood isolates from gastrointestinal cancer patients for the detection of disseminated tumor cells using reverse transcription PCR. *Cancer Res* **57**: 3106–3110

Tallini G, Ghossein RA, Emanuel J, Gill J, Kinder B, Dimich AB, Costa J, Robbins R, Burrow GN and Rosai J (1998) Detection of thyroglobulin, thyroid peroxidase, and RET/PTC1 mRNA transcripts in the peripheral blood of patients with thyroid disease. *J Clin Oncol* **16**: 1158–1166

Tezelman S and Clark OH (1995) Current management of thyroid cancer. *Adv Surg* **28**: 191–221

Weber T, Hölting T, Klar E and Herfarth C (1997) The value of scintigraphy for diagnosis in differentiated thyroid cancer is questionable. *Eur J Cancer* **33**: 272–273 (abstract)

Weber T, Ridder R, Willeke F, Lacroix J, Hölting T, Elser H, Klar E, Herfarth C and von Knebel Doeberitz M (1998) Detection of thyroglobulin mRNA in peripheral blood of patients with thyroid cancer. *J Endocrinol Invest* **21**: 12 (abstract)

Weitz J, Kienle P, Lacroix J, Willeke F, Benner A, Lehnert T, Herfarth C and von Knebel Doeberitz M (1998) Dissemination of tumor cells in patients undergoing surgery for colorectal cancer. *Clin Cancer Res* **4**: 343–348

Wyld DK, Selby P, Perren TJ, Jonas SK, Allen-Mersh TG, Wheeldon J and Burchill SA (1998) Detection of colorectal cancer cells in peripheral blood by reverse-transcriptase polymerase chain reaction for cytokeratin 20. *Int J Cancer* **79**: 288–293

*© 2000 Cancer Research Campaign*
